# Metastatic Squamous Cell Carcinoma of the Anus to the Lung Confirmed with Allelotyping

**DOI:** 10.1155/2014/608521

**Published:** 2014-02-09

**Authors:** Rachel Roth, Susan Moffatt-Bruce, Marino E. Leon

**Affiliations:** ^1^Department of Pathology, The Ohio State University Wexner Medical Center, 410 West 10th Avenue, Columbus, OH 43210, USA; ^2^Division of Thoracic Surgery, The Ohio State University Wexner Medical Center, 410 West 10th Avenue, Columbus, OH 43210, USA; ^3^Head and Neck Pathology and Cytopathology, Department of Anatomic Pathology, H. Lee Moffitt Cancer Center and Research Institute, University of South Florida, Tampa, FL 33612, USA; ^4^Department of Oncologic Sciences and Department of Pathology and Cell Biology, University of South Florida, Tampa, FL 33620, USA

## Abstract

Histopathologic techniques are insufficient for distinguishing primary squamous cell carcinoma (SCC) from metastatic SCC, which is clinically important. A patient with SCC of the anus was found to also have SCC of the lung, and the question of metastatic versus synchronous primary diseases was raised. Immunohistochemical and hematoxylin and eosin (H&E) staining on sections of tissue could not discriminate between the two entities. Immunostain for p16 and chromogenic *in situ* hybridization for human papillomavirus (HPV) type 16 were positive in both tumors. Additionally, allelotyping for loss of heterozygosity displayed similar findings and confirmed the histopathological impression of anal SCC metastasis to the lung. The patient was treated with palliative chemotherapy instead of additional surgical treatment. When multiple tumors are present, determining metastatic versus synchronous primary tumors is necessary for appropriate treatment. Identification can be achieved using allelotyping for loss of heterozygosity.

## 1. Introduction

Often clinical judgment has to be relied upon to differentiate between primary squamous cell carcinoma (SCC) of the lung and metastatic SCC of the anus. Hematoxylin and eosin (H&E) and immunohistochemical (IHC) staining on biopsy cannot distinguish the two entities. This case report exemplifies a woman with SCC of the anus with confirmation of lung metastasis using human papillomavirus type 16 (HPV-16) chromogenic *in situ* hybridization (CISH) and allelotyping for loss of heterozygosity (LOH). To the best of our knowledge, this is the first case of anal SCC metastatic to the lung identified with p16 immunostaining, HPV type, and allelotyping in the literature.

## 2. Materials, Methods, and Results

**Figure 1:**
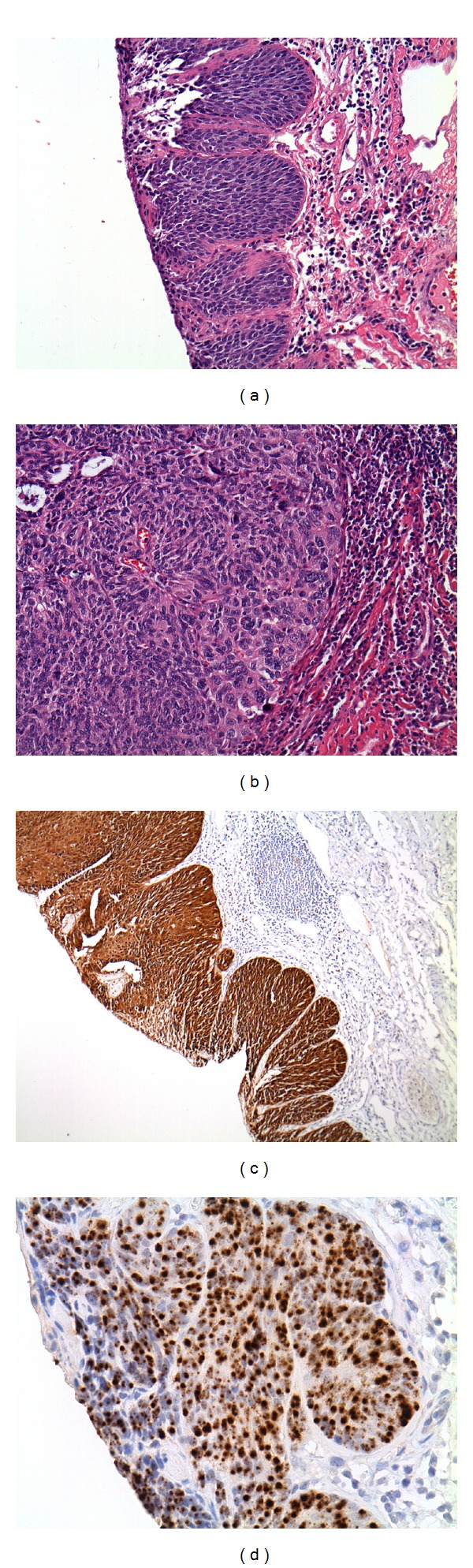
Hematoxylin and eosin staining on anal fissure excision at 5x magnification (a) and 20x magnification (b). Immunohistochemical staining for p16 (c) and CISH for HPV-16 (d) were both positive.

The patient is a 75-year-old woman diagnosed with a stage IV SCC of the anus. The patient reported hemorrhoidal problems which began to worsen in July 2003. A colonoscopy revealed the presence of grade 2 internal hemorrhoids, an anterior anal fistula, and a posterior anal papilloma. A fistulotomy and polypectomy were performed. The pathology report on the papilloma was unremarkable. She continued to have rectal bleeding and pain at the procedure site until a repeat colonoscopy was performed in March 2004, which revealed an anal fissure that was excised. At that time, the pathology report revealed an invasive poorly differentiated SCC with negative margins (Figure 1).

Her past medical history is significant for osteoarthritis and depression, and she has never used tobacco. She underwent a computed tomography (CT) scan of the abdomen and pelvis, which reported mild wall thickening of the anorectal region without lymphadenopathy or extension of the tumor into the perirectal soft tissue. Chemotherapy and radiation therapy were both initiated, and she successfully completed both regimens 3 months later. During a follow-up appointment, a digital rectal exam revealed no palpable defects and complete healing of the prior procedure, and a colonoscopy performed in November 2004 was unremarkable. A CT scan of the chest performed within two years revealed an enlarging left lower lobe lung lesion. The lesion was positive on positron emission tomography (PET) scan, which was concerning for malignancy. The patient underwent a bronchoscopy and a left video-assisted thoracoscopy with wedge resection of the left lower lobe lung lesion in October 2009. The pulmonary tissue was formalin fixed and paraffin embedded using conventional methods. Four-micrometer sections of tissue were stained with H&E. Histopathologic examination of the lung lesion revealed poorly differentiated SCC with extensive necrosis; the tumor showed similar histopathologic findings to the lesion in the anus. Immunohistochemical staining of sections of the pulmonary and anal lesions was positive for p16 (Figure 1).

**Figure 2:**
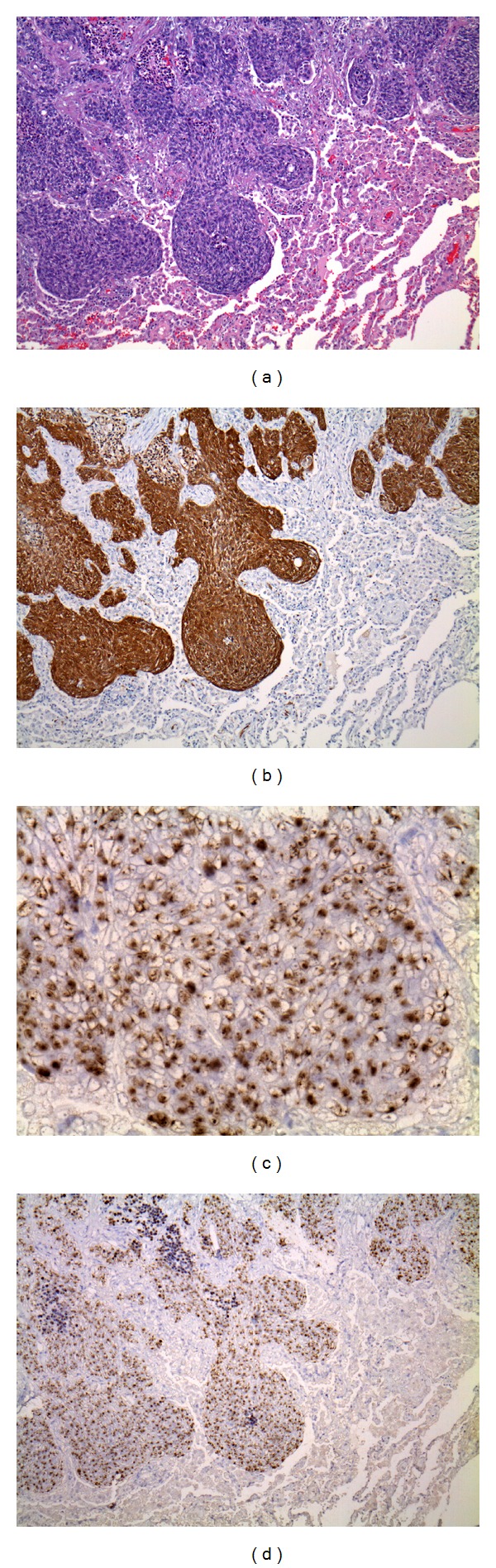
Hematoxylin and eosin stain (a) and p16 immunohistochemical stain (b) on sections of lesion in left lower lobe wedge resection. CISH for human papillomavirus type 16 is seen at 400x original magnification (c) and 100x original magnification (d).

To determine that the pulmonary SCC was a metastasis from the prior anal SCC and not a *de novo* primary lung cancer, a CISH study for HPV type 16 (HPV-16) was performed on the anal fistulectomy specimen and the lung wedge resection tissue. The CISH assay was performed with a HPV-16 specific probe. This test was performed at The Ohio State University Department of Pathology Core Facility, which is accredited by the College of American Pathologists (CAP) and by the Clinical Laboratory Improvement Amendments (CLIA). The slides for both cases were examined using light microscopy. A hybridization of the probe with integrated HPV in the tumor was defined as a positive signal demonstrated by dark brown dot-like staining in the nucleus of the tumor cells. Appropriate positive and negative controls were run with the samples. Internal negative controls were also reviewed for each tissue section. Both the anal and the pulmonary squamous cell carcinoma demonstrated positive hybridization for HPV-16. Additionally, both tumors had identical histopathologic findings (Figure 2).

However, a definitive metastatic disease could not be distinguished from metachronous primary tumors; therefore, allelotyping using LOH was undertaken. Microdissection for the allelotyping was performed on conventional 4-micrometer-thick unstained histologic sections of formalin-fixed and paraffin-embedded tissue. Multiple microdissection targets were acquired from each tumor to determine intratumoral heterogeneity. Allelotyping consisted of both DNA sequencing for specific oncogene point mutation detection (k-ras-2) and a broad panel LOH cancer-associated marker. LOH was quantitative (polymerase chain reaction/capillary electrophoresis), allowing the detection of marker LOH and defining specific allele copy affected by imbalance and the temporal sequence of mutational acquisition over time. The marker panel targeted 1p, 3p, 5q, 9p, 10q, 17p, 17q, 18q, 21q, and 22q. Thresholds for accurate discrimination between true genomic loss and fluctuations from nucleic acid amplification were established. When one allele was absent in a microdissected neoplastic tissue section, this was recognized as a genomic deletion. Minor degrees of allele peak variation were corrected with algorithms dividing allelic ratios of tumor cells by those of nontumor cells. Normal ranges for each pair of alleles of given markers were based on a representative number of normal specimens from patients with no known neoplastic disease, which were used as controls.

Thresholds for minimal significant allelic loss were defined and applied. The proportion of cells with mutations in an individual microdissected sample was also assessed. To diagnose metastatic disease, determination of concomitant LOH affecting the same allele copy between the 2 tumor sites was necessary. Diagnosing metachronous primary tumors depended upon complete discordance with respect to temporal sequence of acquisition and/or specific allele copy involvement. The tumors are distinguished as primary or metastatic based on 3 levels of concordance. These include marker-affected tumors being considered concomitant if 50% or more of the same markers are mutated, the same gene copy is affected, and the temporal sequence of mutation is similar.

**Figure 3:**
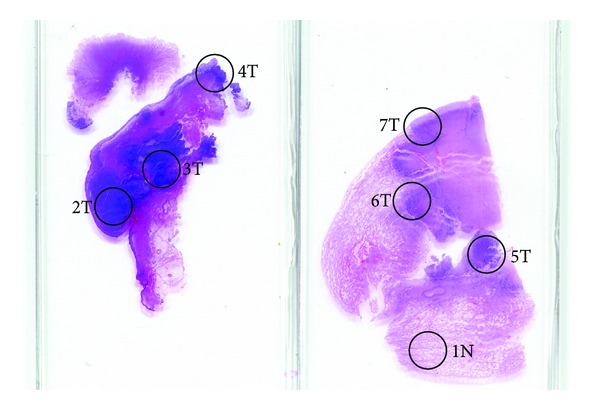
Hematoxylin and eosin stained sections of tumors from the anal canal (left) and lung tumor (right) showing the sampled foci. Anal SCC sampled foci: 2T, 3T, and 4T; lung SCC sampled foci: 5T, 6T, and 7T; and lung normal: 1N for the loss of heterozygosity (LOH) allelotyping performed on lung tissue.

In the patient described in this case report, both tumors had identical allelic imbalance of 3p and 5q with the same allele copy affected in each specimen. The lung tumor had an additional 17p LOH. These data confirmed a metastatic anal SCC to the lung. The patient is currently clinically stable without signs of recurrent disease (Figure 3).

## 3. Discussion

Squamous cell carcinoma of the anal canal is a rather rare phenomenon accounting for only 4% of all anorectal tumors. The majority of these tumors present as fissures, hemorrhoids, or anorectal fistulae [1]. Lung metastases are rare in this neoplasm, and those that do metastasize to the thoracic cavity do so usually after local or inguinal recurrence [2]. A few cases of anal SCC metastatic to the lung are reported in the literature; however, this is the first case of an anal squamous cell carcinoma identified with p16 immunostaining, *in situ* hybridization for HPV-16, and allelotyping. The microscopic examination of H&E stained slides of tissue will allow pathologists to categorize the tumor into general histopathologic groups, and immunohistochemical staining can help to further identify tumor origin by highlighting protein markers that are tissue-specific. However, unlike adenocarcinoma, there are no useful immunohistochemical markers that can determine the specific site of origin for SCC at this time. Also, a disadvantage of immunohistochemistry includes the loss of these protein markers in poorly differentiated/high grade tumors [3, 4]. Cytokeratin is an intermediate filament protein located in the cytoskeleton of normal epithelium as well as epithelial neoplasms. There are at least 20 different cytokeratin proteins that have been discovered to date. In the lung, expression of cytokeratin 7 can be seen in 23% of primary SCC, and expression of cytokeratin 5/6 can be seen in 100% of primary SCC. SCC of the anal canal can show expression of cytokeratin 7 and cytokeratin 5/6 as well [5, 6]. Another immunohistochemical marker that is expressed in both lung and anal SCC is p63, which is expressed in the majority (more than 80%) of SCC from various regions [6]. The marker p16 is a tumor suppressor protein highly expressed in HPV-associated cervical carcinomas and dysplasia as well as anorectal squamous cell carcinoma and dysplasia [4]. Furthermore, our patient was shown to have p16 positivity and CISH for HPV-16 positive SCC in the lung. As p16 and CISH for HPV-16 were not originally performed in the anal specimen, these stains were performed on the anal tumor in conjunction with the lung specimen. The anal tumor showed strong p16 expression and positive reaction with CISH HPV-16. A discrepancy in staining patterns would have been useful in differentiating metastatic versus primary disease.

The metastatic origin of the lung lesion is very important to differentiate from primary lung carcinoma for staging purposes, prognosis, and ultimately treatment modalities. Therefore, in this case, more advanced techniques had to be utilized to determine the site of origin, including CISH for HPV-16 and LOH allelotyping.

Human papillomaviruses are small DNA viruses that infect cutaneous or mucosal epithelium. HPV types 16 and 18 have been found to cause over 99% of cervical carcinomas, and these two types have been linked to carcinoma development in all internal organs with the exception of the heart and kidney [7].

If the anal SCC was positive for HPV type 16 and the pulmonary SCC was negative, that would indicate that the pulmonary SCC is a metachronous primary instead of metastasis. In this case, both the anal SCC and the lung were positive for p16 and HPV-16. These findings were very suggestive of a metastatic origin for the lung tumor. However, as HPV-16 is the most frequently detected HPV type in as many as 46% of primary lung SCC, the p16 and HPV-16 studies did not prove unequivocally that the pulmonary SCC was a metastasis from the anal SCC. These findings also did not prove that both tumors had a common clonal origin. Therefore, allelotyping for LOH was necessary to determine site of origin of the pulmonary tumor.

Most commonly, carcinomas can have areas on chromosomes in which the DNA is altered or sustains a LOH. Allelotyping is a process that involves withdrawing and amplifying the DNA to search for patterns of LOH or alteration [8]. The allelotype seen in both of our patient's tumors was similar, meaning the tumors originated from the same clone. This was strong evidence in support of metastasis from the anal SCC to the pulmonary parenchyma years after the original carcinoma occurred. If the allelotypes of each tumor had been dissimilar, they would have been considered separate entities or metachronous primary tumors.

## 4. Conclusion

H&E and immunohistochemical staining are very important in the process of identifying carcinoma. However, when treatment and prognosis are uncertain because the site of origin for a tumor is unknown, other techniques such as HPV typing by CISH and allelotyping for LOH can be very useful. These techniques can help to avoid misdiagnosis and mistreatment of neoplasia of metachronous tumors with the same histopathologic morphology.

## References

[1] Khysh VI, Timofeev IM, Rottenberg VI (1983). Squamous cell cancer of the anal canal. *Voprosy Onkologii*.

[2] Gerard J-P, Ayzac L, Hun D (1998). Treatment of anal canal carcinoma with high dose radiation therapy and concomitant fluorouracil-cisplatinum. Long-term results in 95 patients. *Radiotherapy and Oncology*.

[3] Park JM, Jung CK, Choi YJ (2010). The use of an immunohistochemical diagnostic panel to determine the primary site of cervical lymph node metastases of occult squamous cell carcinoma. *Human Pathology*.

[4] Balachandra B, Marcus V, Jass JR (2007). Poorly differentiated tumours of the anal canal: a diagnostic strategy for the surgical pathologist. *Histopathology*.

[5] Marson VJ, Mazieres J, Groussard O (2004). Expression of TTF-1 and cytokeratins in primary and secondary epithelial lung tumours: correlation with histological type and grade. *Histopathology*.

[6] Kaufmann O, Fietze E, Mengs J, Dietel M (2001). Value of p63 and cytokeratin 5/6 as immunohistochemical markers for the differential diagnosis of poorly differentiated and undifferentiated carcinomas. *American Journal of Clinical Pathology*.

[7] Aguayo F, Castillo A, Koriyama C (2007). Human papillomavirus-16 is integrated in lung carcinomas: a study in Chile. *British Journal of Cancer*.

[8] Seward SM, Richardson DL, Leon ME, Zhao W, Cohn DE, Hitchcock CL (2009). Metastatic squamous cell carcinoma of the vulva to the lung confirmed with allelotyping. *International Journal of Gynecological Pathology*.

